# Sardjito Cardiovascular Intensive Care Score as an Alternative to Mayo Cardiac Admission Risk Score for Predicting Mortality in Cardiovascular Intensive Care Patients

**DOI:** 10.14740/cr2199

**Published:** 2026-06-05

**Authors:** Nadia Luthfia Adani, Hendry Purnasidha Bagaswoto, Irsad Andi Arso

**Affiliations:** a Department of Cardiology and Vascular Medicine, University of Gadjah Mada, Yogyakarta, Indonesia; b Cardiology Division, Sardjito Hospital, Yogyakarta, Indonesia

**Keywords:** Cardiac intensive care, Cardiovascular patients, SCIENCE, M-CARS, CICU

## Abstract

**Background:**

The cardiovascular intensive care unit (CICU) has evolved into a multidisciplinary unit managing critically ill patients with high mortality rates. In 2022, the Sardjito Cardiovascular Intensive Care (SCIENCE) score introduced seven simple parameters assessed within the first 24 h to predict CICU mortality. The Mayo Cardiac Admission Risk Score (M-CARS) is a well-established tool for predicting CICU mortality but requires specialized laboratory tests. This study compared the predictive performance of the SCIENCE score and M-CARS and validated SCIENCE at its development site.

**Methods:**

This retrospective cohort study was conducted between February 2022 and September 2024 and included CICU patients at Sardjito Hospital. Outcomes assessed were CICU and in-hospital mortality. Predictive performance was evaluated by accuracy, discrimination, and calibration (Hosmer–Lemeshow test).

**Results:**

Over 50% of the initial participants were excluded due to missing M-CARS data (particularly the anion gap), leaving 1,503 eligible patients. M-CARS showed good predictive performance for CICU mortality (accuracy 70.9%, area under the curve (AUC) 0.804, P = 0.685) and in-hospital mortality (accuracy 72.2%, AUC 0.797, P = 0.303). The SCIENCE score also showed good predictive performance for CICU mortality (accuracy 63.7%, AUC 0.775, P = 0.059) and in-hospital mortality (accuracy 65.8%, AUC 0.767, P = 0.352). Acute stroke (hemorrhagic and non-hemorrhagic) was associated with higher CICU and in-hospital mortality (P < 0.05).

**Conclusion:**

Both the SCIENCE score and M-CARS provide acceptable predictive performance for CICU patients. However, as M-CARS has limited applicability in some settings, the SCIENCE score may serve as a more practical alternative.

## Introduction

The cardiovascular intensive care unit (CICU) is originally a coronary heart care unit; however, it has evolved into an integrated multidisciplinary unit for cardiovascular patients [1, 2]. Several studies have demonstrated an increase in age, comorbidities, prevalence of noncardiac diseases, and procedures among patients admitted in the CICU. A shift in the patient population from acute myocardial infarction to those with circulatory failure and multi-organ dysfunction has been observed, leading to high complexity and mortality [3–7]. Reported CICU mortality rates range from 5.9% in Thailand to 9.2% in the United States [8, 9]. At Sardjito Hospital Indonesia, the mortality rate of CICU patients in 2017 reached 9.2% [10]. These high mortality rates highlight the significance of effective risk stratification to aid in patient triage, optimize resource allocation, and predict outcomes [1].

Several ICU risk scores exist; however, CICU mortality-specific tools remain limited [2, 3, 11]. The Mayo Cardiac Admission Risk Score (M-CARS), introduced in 2019, is the only well-known mortality risk assessment score for CICU patients. The M-CARS assesses the following seven predictive factors for mortality within the first 24 h of CICU admission: admission diagnosis of cardiac arrest, admission diagnosis of shock, admission diagnosis of respiratory failure, Braden skin score, blood urea nitrogen (BUN) level, anion gap (AG) value, and red cell distribution width. The M-CARS has demonstrated excellent performance in the United States CICU patient population with an area under the curve (AUC) of 0.864 (95% confidence interval (CI), 0.842–0.886), sensitivity of 75%, and specificity of 73% [8, 9]. An external validation study revealed that M-CARS of > 6 points were associated with high in-hospital mortality rates (AUC, 0.840) [9].

In developed countries, AG analysis is routine but frequently omitted in resource-limited settings owing to cost [12]. A study on the M-CARS in Thailand has revealed an interesting issue, reporting that among 1,988 participants, almost a quarter did not have AG data within 24 h of initial CICU admission [9]. The pioneering M-CARS study by Jentzer has reported that the most frequently unavailable M-CARS data within the first 24 h of admission was the AG and was absent in 10.8% of the cases [8]. These studies emphasize the significance of alternative CICU scoring systems with more applicable parameters.

In 2022, a new scoring system, the Sardjito Cardiovascular Intensive Care (SCIENCE) score, was introduced for predicting mortality in CICU patients. This score was developed using the following seven predictor variables within the first 24 h of admission: female sex, acute heart failure, hemodynamic instability, pneumonia, creatinine level > 1.5 mg/dL, tricuspid annular plane systolic excursion < 17 mm, and the use of mechanical ventilation. The SCIENCE score had an AUC of 0.75, with a sensitivity of 75% and specificity of 65%. The in-hospital mortality rate increases as the SCIENCE score increases [13, 14]. As the SCIENCE score does not require AG data, it is anticipated to be a simpler tool. This study aimed to compare the predictive performance (discrimination power, accuracy, and calibration power) of the SCIENCE score and M-CARS. Moreover, this study serves as an internal validation of the SCIENCE score within its original development site.

## Materials and Methods

### Design and participants

This was an observational analytical study with a retrospective cohort design. We used an existing SCIENCE registry database of adult patients (≥ 18 years old) admitted to the CICU at Sardjito Hospital Yogyakarta, Indonesia, from February 1, 2022 to September 30, 2024. Patients admitted or hospitalized in the CICU were included in this study. The exclusion criteria in this study were consistent with those applied in the pioneering M-CARS and SCIENCE studies, including post-cardiac surgery patients, patients using extracorporeal membrane oxygenation devices, those undergoing monitoring following elective cardiac procedures, and those who do not have complete research variable data within the first 24 h of hospital CICU admission. Three independent reviewers manually extracted the variables required for calculating the SCIENCE score and M-CARS from electronic medical records and laboratory reports using a standardized data collection form. Any discrepancies among reviewers were resolved through consensus to ensure data reliability. Each patient mortality risk was calculated using the M-CARS (Supplementary Material 1, cr.elmerpub.com) and SCIENCE score (Supplementary Material 2, cr.elmerpub.com), similar to the pioneering studies [8, 13]. According to the pioneering studies, patients have a high risk of mortality when they have a SCIENCE score of ≥ 3 or M-CARS of ≥ 4 [8, 13]. All-cause mortality, defined as death from any cause, was used as the outcome measure in this study and was assessed during the CICU stay (CICU mortality) and the overall hospitalization period (in-hospital mortality).

### Statistical analysis

Numeric data were summarized as means ± standard deviations or medians (min–max), depending on the distribution. Categorical data were expressed as frequencies and percentages. Normality was tested using the Kolmogorov–Smirnov test (P > 0.05 = normal). Bivariate analysis was analyzed using the Chi-square test. To determine the factors influencing mortality, a multivariate analysis was conducted with a logistic regression test. The predictive performance in this study was determined by examining accuracy, discrimination, and calibration. The accuracy was analyzed using a 2 × 2 table to calculate the sensitivity, specificity, positive predictive value (PPV), negative predictive value (NPV), positive likelihood ratio, and negative likelihood ratio. Discrimination was analyzed using a receiver operating characteristic (ROC) curve. An AUC value of > 0.70 indicates good and acceptable discrimination power [15, 16]. Using the Hosmer–Lemeshow test, calibration analysis of the SCIENCE score and M-CARS for CICU patient mortality was considered statistically significant at P < 0.05.

## Results

### Baseline characteristics

**Figure 1:**
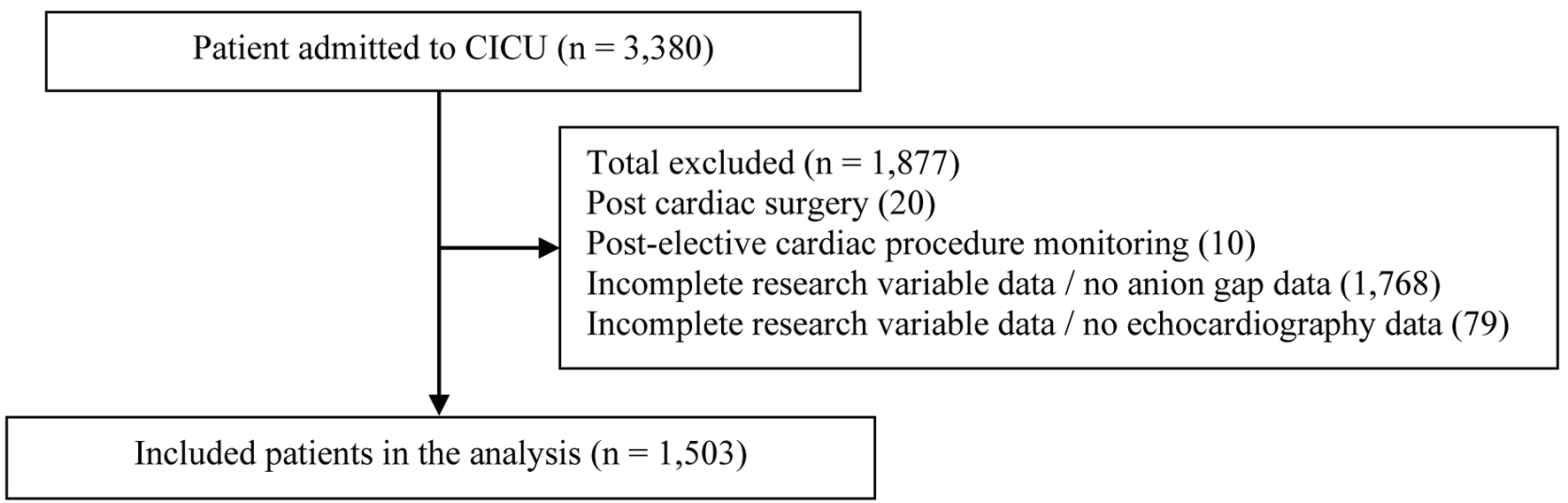
Flow diagram demonstrating inclusion and exclusion criteria. Most of subjects were excluded due to incomplete data of anion gap. CICU: cardiovascular intensive care unit.

This study included 3,380 patients with a primary diagnosis of acute cardiovascular disease admitted to the CICU of Sardjito Hospital from January 2022 to September 2024. During the hospitalization period, 601 (17.8%) patients died. However, 1,877 participants were excluded after applying the exclusion criteria, resulting in 1,503 participants. The excluded participants comprised 20 patients with a post-cardiac surgery diagnosis, either valve surgery or heart bypass surgery; 10 patients were monitoring patients following elective heart procedures; 1,768 patients did not have AG value data; and 79 patients did not have echocardiography data in the first 24 h of admission (Fig. 1.)

The baseline characteristics of the study and bivariate tests on CICU mortality and in-hospital mortality are presented in Table 1. Regarding demographic characteristics, the median age of the participants was 62 (18–99) years, with majority of them in the ≥ 60-year-old category. Participants aged ≥ 60 years exhibited a significantly higher proportion of hospital deaths. Males had a higher proportion than females (66.1% vs. 33.9%) [9].

**Table 1:** Basic Characteristics of the Study Population

Variable	Total (n = 1,503)	CICU mortality		P value	In-hospital mortality		P value
		Yes (n = 474)	No (n = 1,029)		Yes (n = 566)	No (n = 937)	
Gender				0.033*			0.057
Men, n (%)	993 (66.1%)	295 (62.2%)	698 (67.8%)		357 (63.1%)	636 (67.9%)	
Women, n (%)	510 (33.9%)	9 (37.8%)	331 (32.2%)		209 (36.9%)	301 (32.1%)	
Age (median)	62 (18–99)			0.444	63 (20–94)	61 (18–99)	0.031*
≥ 60 years	879 (58.5%)	284 (59.9%)	595 (57.8%)		351 (62%)	528 (56.4%)	
< 60 years	624 (41.5%)	190 (40.1%)	434 (42.2%)		215 (38%)	409 (43.6%)	
Admission diagnosis							
Acute heart failure, n (%)	623 (41.5%)	206 (43.5%)	417 (40.5%)	0.283	252 (44.5%)	371 (39.6%)	0.06
Shock/hemodynamic instability, n (%)	642 (42.7%)	322 (67.9%)	320 (31.1%)	0.001*	366 (64.7%)	276 (29.5%)	0.001*
Acute coronary syndrome, n (%)	999 (66.4%)	307 (64.5%)	692 (67.2%)	0.346	352 (62%)	647 (69%)	0.06
STEMI	708 (70.9%)	222 (72.3%)	486 (70.2%)		246 (69.9%)	462 (71.4%)	
NSTEMI	273 (27.3%)	83 (27%)	190 (27.5%)		103 (29.3%)	170 (26.3%)	
UAP	18 (1.8%)	2 (0.7%)	16 (2.3%)		3 (0.9%)	15 (2.3%)	
Arrhythmia, n (%)	352 (23.4%)	108 (22.8%)	244 (23.7%)	0.693	127 (22.4%)	225 (44.5%)	0.486
Vascular emergency, n (%)	61 (4.1%)	24 (5.1%)	37 (3.6%)	0.180	30 (5.3%)	31 (3.3%)	0.058
Cardiac arrest, n (%)	203 (13.5%)	103 (21.7%)	100 (9.7%)	0.001*	112 (19.8%)	91 (9.7%)	0.001*
Comorbid							
Respiratory failure, n (%)	422 (28.1%)	258 (54.4%)	164 (15.9%)	0.001*	286 (50.5%)	136 (14.5%)	0.001*
Pneumonia, n (%)	358 (23.8%)	136 (28.7%)	807 (78.4%)	0.003*	166 (29.3%)	192 (20.5%)	0.001*
Urinary tract infection, n (%)	157 (10.4%	56 (11.8%)	101 (9.8%)	0.239	71 (12.5%)	86 (9.2%)	0.039
Renal chronic disease, n (%)	60 (4%)	17 (3.6%)	43 (4.2%)	0.586	25 (4.1%)	37 (3.8%)	0.702
Sepsis, n (%)	52 (3.3%)	26 (5.5%)	22 (2.1%)	0.001*	31 (5.5%)	17 (1.8%)	0.001*
Acute non-hemorrhagic stroke, n (%)	84 (5.6%)	37 (7.8%)	47 (4.6%)	0.011*	47 (8.3%)	37 (3.9%)	0.001*
Hemorrhagic stroke, n (%)	4 (0.3%)	4 (0.8%)	0 (0%)	0.010*	4 (0.7%)	0 (0%)	0.020*
Invasive ventilation usage, n (%)	384 (25.5%)	264 (55.7%)	120 (11.7%)	0.001*	287 (50.7%)	97 (10.4%)	0.001*
Non-invasive ventilation usage, n (%)	61 (4.1%)	23 (4.9%)	38 (3.7%)	0.290	28 (4.9%)	33 (3.5%)	0.175
Echocardiography							
LVEF				0.001*			0.001*
≤ 40%	699 (46.5%)	261 (55.1%)	438 (42.6%)		298 (52.7%)	401 (42.8%)	
41–49%	262 (17.4%)	77 (16.2%)	185 (18%)		174 (18.6%)	262 (17.4%)	
≥ 50%	542 (36.1%)	136 (28.7%)	406 (39.5%)		180 (31.8%)	362 (38.6%)	
TAPSE				0.001*			0.001*
< 17 mm	588 (39.1%)	240 (50.6%)	348 (33.8%)		270 (47.7%)	318 (33.9%)	
≥ 17 mm	915 (60.9%)	234 (49.4%)	681 (66.2%)		296 (52.3%)	619 (66.1%)	
Laboratory							
Creatinine (mg/dL)				0.001*			0.001*
≥ 1.5 mg/dL	810 (53.9%)	340 (71.7%)	470 (45.7%)		401 (70.8%)	409 (43.6%)	
< 1.5 mg/dL	693 (46.1%)	134 (28.3%)	559 (54.3%)		165 (29.2%)	528 (56.4%)	
BUN (mg/dL)				0.001*			0.001*
> 23 mg/dL	856 (57%)	349 (73.6%)	507 (49.3%)		418 (73.9%)	438 (46.7%)	
≤ 23 mg/dL	647 (43%)	125 (26.4%)	522 (50.7%)		148 (26.1%)	499 (53.3%)	
RDW (%)				0.001*			0.001*
> 14.3	556 (37%)	208 (43.9%)	348 (33.8%)		260 (45.9%)	296 (31.6%)	
≤ 14.3	947 (63%)	266 (56.1%)	681 (66.2%)		306 (54.1%)	641 (68.4%)	
Anion gap				0.001*			0.001*
> 14	835 (55.6%)	350 (73.8%)	485 (47.1%)			435 (46.4%)	
≤ 14	668 (44.4%)	124 (26.2%)	544 (52.9%)			502 (53.6%)	
Lactate (median)	2.2 (0.32–19)	3.25 (0.32–19.4)	1.93 (0.33–19.4)	0.001*		1.9 (0.33–19)	0.001*
Others							
The use of circulatory support device/intra-aortic balloon pump, n (%)	17 (1.1%)	9 (1.9%)	8 (0.8%)	0.056	10 (1.8%)	7 (0.7%)	0.070
Renal replacement therapy, n (%)	73 (4.9%)	38 (8%)	35 (3.4%)	0.001*	47 (8.3%)	26 (2.8%)	0.001*
Braden skin score				0.001*			0.001*
≤ 12	350 (23.3%)	244 (51.5%)	106 (10.3%)		271 (47.9%)	79 (8.4%)	
13–15	507 (33.7%)	199 (25.1%)	388 (37.7%)		147 (26%)	360 (38.4%)	
> 15	646 (43%)	111 (23.4%)	535 (52%)		148 (26.1%)	498 (53.1%)	
SCIENCE score				0.001*			0.001*
High risk (≥ 3)	858 (57.1%)	393 (82.9%)	465 (45.2%)		455 (80.4%)	403 (43%)	
Low risk (< 3)	645 (42.9%)	81 (17.1%)	564 (54.8%)		111 (19.6%)	534 (57%)	
M-CARS score				0.001*			0.001*
High risk (≥ 4)	715 (47.6%)	376 (79.3%)	339 (32.9%)		432 (76.3%)	283 (30.2%)	
Low risk (< 4)	788 (52.4%)	98 (20.7%)	690 (67.1%)		134 (23.7%)	654 (69.8%)	
Outcomes							
CICU mortality	474 (31.5%)						
Hospital mortality	566 (37.7%)						

*Statistically significant difference (P < 0.05). BUN: blood urea nitrogen; CICU: cardiovascular intensive care unit; LVEF: left ventricle ejection fraction; M-CARS: Mayo Cardiac Admission Risk Score; RDW: red cell distribution width; SCAI: Society for Cardiovascular Angiography and Interventions; SCIENCE: Sardjito Cardiovascular Intensive Care; TAPSE: tricuspid annular plane systolic excursion.

In this study, the CICU and in-hospital mortality rates were 31.5% (n = 474) and 37.7% (n = 566), respectively. This study also revealed noncardiovascular comorbidities, including respiratory failure (28.1%), pneumonia (23.8%), urinary tract infection (10.4%), chronic kidney disease (4%), sepsis (3.3%), acute nonhemorrhagic stroke (5.6%), and hemorrhagic stroke (0.3%). The results indicate high noncardiovascular comorbidity rates among CICU patients even on the first day of admission.

**Figure 2:**
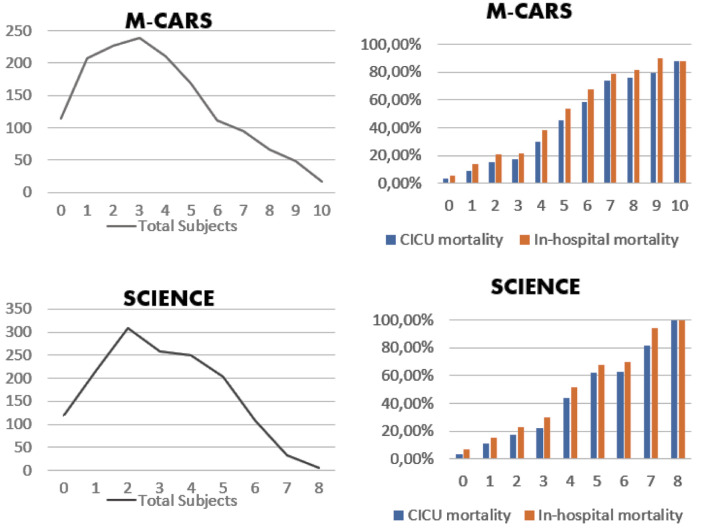
Distribution of SCIENCE and M-CARS score. The mortality rate will increase along with the increase in SCIENCE and M-CARS points. CICU: cardiovascular intensive care unit; M-CARS: Mayo Cardiac Admission Risk Score; SCIENCE score: Sardjito Cardiovascular Intensive Care score.

The distribution of participants in the M-CARS and SCIENCE score, as well as mortality proportions, is described in Figure 2. The highest M-CARS (10 points) reached 87.5% of CICU and in-hospital mortality. In comparison, the highest SCIENCE score (8 points) reached 100% of CICU and in-hospital mortality in our study. The mortality rate increases as the SCIENCE score and M-CARS increase. The participant distribution in the M-CARS and SCIENCE score was relatively proportional (Fig. 2). This finding differed from those of similar studies conducted in Thailand, wherein approximately 50% of the participants had an M-CARS of 0 or 1, whereas < 2% had an M-CARS of 7 or 8 [9]. The pioneering M-CARS study, including the Mayo database, showed approximately 10% of patients having a score of 7–10 [8].

### Predictive performance

In this study, the predictive ability of the M-CARS and SCIENCE score was evaluated in terms of accuracy, discrimination quality, and calibration quality. The accuracy of both scores was assessed against predicting mortality events in the CICU and in-hospital (Table 2). The results revealed that the M-CARS had a sensitivity, specificity, PPV, NPV, and accuracy of 72.32%, 67%, 52.59%, 87.56%, and 70.9%, respectively, for CICU mortality outcomes. The SCIENCE score has a sensitivity, specificity, PPV, NPV, and accuracy of 82.91%, 54.81%, 45.8%, 87.44%, and 63.67%, respectively, for CICU mortality outcomes. For in-hospital mortality outcomes, the M-CARS showed a sensitivity, specificity, PPV, NPV, and accuracy of 76.3%, 69.8%, 60.4%, 82.9%, and 72.2%, respectively. For in-hospital mortality outcomes, the SCIENCE score exhibited a sensitivity, specificity, PPV, NPV, and accuracy of 80.39%, 56.99%, 53.03%, 82.79%, and 65.8%, respectively.

**Table 2:** Accuracy Test of SCIENCE and M-CARS Upon CICU and Hospital Mortality Outcomes

	Score category		Death	Alive	Sn (%)	Sp (%)	PPV (%)	NPV (%)	Accuracy (%)
CICU outcomes	M-CARS	High risk (≥ 4)	376	339	72.32	67.07	52.59	87.56	70.92
		Low risk (< 4)	98	690					
	SCIENCE	High risk (≥ 3)	393	465	82.91	54.81	45.80	87.44	63.67
		Low risk (< 3)	81	564					
Hospital outcomes	M-CARS	High risk (≥ 4)	432	283	76.33	69.80	60.42	82.99	72.26
		Low risk (< 4)	134	654					
	SCIENCE	High risk (≥ 3)	455	403	80.39	56.99	53.03	82.79	65.80
		Low risk (< 3)	111	534					

CICU: cardiovascular intensive care unit; M-CARS: Mayo Cardiac Admission Risk Score; NPV: negative predictive value; PPV: positive predictive value; SCIENCE score: Sardjito Cardiovascular Intensive Care score; Sn: sensitivity; Sp: specificity.

**Figure 3:**
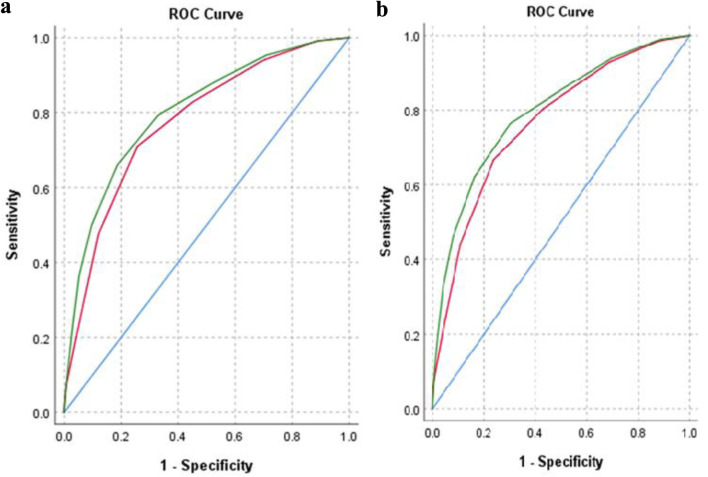
M-CARS and SCIENCE ROC curves of (a) CICU mortality outcome and (b) in-hospital mortality outcome. Analysis of discrimination quality with the M-CARS (green line) and SCIENCE score (red line) have significant values (P < 0.001). CICU: cardiovascular intensive care unit; M-CARS: Mayo Cardiac Admission Risk Score; ROC: receiver operating characteristic; SCIENCE score: Sardjito Cardiovascular Intensive Care score.

To determine the AUC and the C statistic value, an analysis of discrimination power with ROC curve was performed. In the curve depicted in Figure 3a and Table 3, regarding CICU mortality outcomes, the M-CARS has an AUC of 0.804 (95% CI, 0.780–0.828; P < 0.001), whereas the SCIENCE score has an AUC of 0.775 (95% CI, 0.751–0.800; P < 0.001). In Figure 3b and Table 3, regarding in-hospital mortality outcomes, the M-CARS has an AUC of 0.797 (95% CI, 0.773–0.820; P < 0.001), whereas the SCIENCE score has an AUC of 0.767 (95% CI, 0.742–0.791; P < 0.001).

**Table 3:** Discrimination Quality Test of SCIENCE and M-CARS

	Variable	AUC	P	95% CI	
				Lower bound	Upper bound
CICU mortality	M-CARS	0.804	< 0.001*	0.780	0.828
	SCIENCE	0.775	< 0.001*	0.751	0.800
In-hospital mortality	M-CARS	0.797	< 0.001*	0.773	0.820
	SCIENCE	0.767	< 0.001*	0.742	0.791

*Statistically significant difference (P < 0.05). AUC: area under the curve; CI: confidence interval; CICU: cardiovascular intensive care unit; M-CARS: Mayo Cardiac Admission Risk Score; SCIENCE score: Sardjito Cardiovascular Intensive Care score.

**Figure 4:**
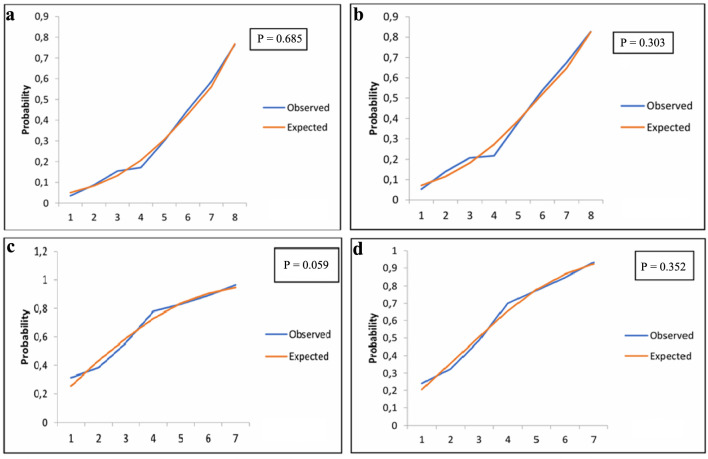
(a) Calibration plot M-CARS towards the CICU mortality outcome. (b) Calibration plot M-CARS towards the in-hospital mortality outcome. (c) Calibration plot SCIENCE towards the CICU mortality outcome. (d) Calibration plot SCIENCE towards the in-hospital mortality outcome. The Hosmer–Lemeshow test (calibration plots) shows that the expected mortality value (red line) and the observed mortality value (blue line) do not differ significantly (P > 0.05) in the figure above. CICU: cardiovascular intensive care unit; M-CARS: Mayo Cardiac Admission Risk Score; SCIENCE score: Sardjito Cardiovascular Intensive Care score.

In this study, the calibration of the M-CARS toward the CICU mortality outcome had a P-value of 0.685 (Fig. 4a), whereas that of the M-CARS toward the hospital mortality outcome had a P-value of 0.303 (Fig. 4b). The calibration of the SCIENCE score toward the CICU mortality outcome had a P-value of 0.059 (Fig. 4c), whereas that of the SCIENCE score toward the hospital mortality outcome had a P-value of 0.352 (Fig. 4d).

## Discussion

This study demonstrates that both the SCIENCE score and the M-CARS provide acceptable predictive performance for mortality among CICU patients, with the M-CARS showing slightly superior discrimination. However, the SCIENCE score exhibited comparable accuracy and calibration, supporting its validity as a mortality prediction tool. Importantly, the study hypothesis was met: the SCIENCE score can serve as a practical alternative to the M-CARS, particularly in settings where certain laboratory parameters required for M-CARS—such as the AG—are not routinely available. These findings confirm that a simplified scoring system based on readily obtainable clinical and echocardiographic variables can still provide reliable prognostic information in critically ill cardiovascular patients.

Furthermore, this study highlights that the SCIENCE score may be more applicable in resource-limited clinical settings, including hospitals where (ABG) analysis and comprehensive laboratory testing are not routinely performed within the first 24 h of CICU admission. In such environments, reliance on the M-CARS may be limited due to missing data, whereas the SCIENCE score, which utilizes more accessible parameters, offers a more feasible and scalable approach for early risk stratification and clinical decision-making.

Our demographic findings are comparable to several similar studies on CICU patients, wherein most patients admitted to the CICU were males [9, 17–20]. A previous study conducted in the CICU of our hospital in 2019 revealed that the admission diagnosis of acute coronary syndrome (ACS) reached 70% [21]. The most common admission diagnosis among the participants was ACS (66.4%) in the form of STEMI, consistent with the findings in Thailand (64%) [9]. Our center had a decreasing proportion of ACS admission diagnoses over time owing to increasing medical resources. In the United States, a lower proportion of ACS was noted (42.5%) [19]. In our study, the mortality rate was relatively high compared with the CICU mortality rate reported in other studies [17, 19, 22–24]. However, of note, in this study, 1,797 patients were excluded, 93% of whom were excluded owing to missing AG data. At our hospital, AG analysis is only performed on patients with specific indications, not as routine practice; therefore, those examined tend to be more severe, leading to the higher mortality rate reported in this study. Over the past few decades, acute respiratory failure, sepsis, and acute kidney failure have increased in prevalence in the CICU. Recent studies have indicated that 50% of patients treated in the CICU experience at least one of these complications [7, 25].

Bivariate analysis revealed that noncardiovascular comorbidities, including respiratory failure, pneumonia, sepsis, and stroke (both hemorrhagic and nonhemorrhagic), had a significantly higher proportion of CICU and hospital mortality. Significant results from the bivariate test in Table 1 are examined in a multivariate analysis, as shown in Supplementary Material 3 (cr.elmerpub.com). In this study (Supplementary Material 3, cr.elmerpub.com), the M-CARS, SCIENCE score, stroke, and left ventricular ejection fraction ≤ 40% significantly affected CICU mortality outcomes.

Furthermore, this study revealed that noncardiovascular comorbidities in the form of acute stroke significantly influenced CICU and in-hospital mortality in the multivariate analysis. We have not noted similar studies regarding the diagnosis of acute stroke comorbidities in the first 24 h of CICU admission as one of the factors associated with CICU mortality. Stroke is a common post-ACS complication and is associated with increased mortality [26]. In a study conducted in ICUs in China, hemorrhagic stroke-related mortality was associated with systolic blood pressure changes (odd ratio (OR), 0.25) and heart disease (OR, 1.94), whereas nonhemorrhagic stroke-related mortality was linked to systolic blood pressure changes (OR, 0.49) and age (OR, 1.03) [26, 27]. The heart–brain axis refers to the physiological interaction between the cardiovascular and nervous systems. Stroke-related cardiac dysfunction can occur through several mechanisms, including the hypothalamic–pituitary–adrenal axis, inflammatory mediators, microRNA, and microvesicles [28–30].

### Predictive performance comparison

The pioneering and external validity studies of the M-CARS in various centers on the mortality outcomes of patients admitted to the CICU demonstrated relatively consistent results with the present study. In the United States, the AUC of the M-CARS was 0.864, superior to other ICU scoring systems [8]. The M-CARS study in Thailand had an AUC of 0.840, whereas that in Medan, Indonesia had an AUC of 0.93 [9, 18]. In the pioneering SCIENCE study, the AUC was 0.75 [13, 23]. The results of these centers differ because the discrimination of risk scores relies on the distribution of risk variables in the study population. The wider the spread of risk, the higher the score discrimination can be. The SCIENCE score showed slightly lower discrimination than the M-CARS; however, the difference was modest. As no formal statistical comparison between AUCs was performed, the significance of this difference remains uncertain.

A calibration plot assessed the calibration quality, depicting the agreement between observed mortality in the study population and predicted in-hospital mortality obtained from the validation cohort in the original study. To test the calibration, the Hosmer–Lemeshow test was employed. Previous M-CARS studies have reported good calibration results; however, in the original study, the SCIENCE score has not mentioned the calibration results [8, 13]. A P-value of > 0.05 indicates no significant difference between the observed and expected mortality rates by the M-CARS and SCIENCE score. Our study revealed that the M-CARS and SCIENCE score are considered accurate.

Notably, all prognostic scores have limitations and potential biases, making none perfect. Moreover, clinical judgment and individual patient factors play a crucial role in decision-making. Although the M-CARS has exhibited better discrimination power, it strongly relies on the AG variable in special laboratory tests for predicting mortality risk. AG is an essential indicator of patient health status; however, it is not routinely performed on all CICU patients and tends to be performed on patients with higher morbidity. These factors may limit the use and accuracy of the M-CARS in some situations, particularly in developing countries or limited facilities.

This study shows that using the M-CARS in our hospital is challenging. More than 50% of the CICU patient population is excluded from this study owing to missing AG data. This finding is much higher than that of similar studies in developed countries where the AG un-availability is approximately 10% [8]. At our hospital, blood gas analysis is not routinely performed, considering that our institution is one of the advanced referral hospitals in Indonesia. Furthermore, using the M-CARS will be challenging to implement in various hospitals in developing countries or limited facilities. Although the SCIENCE score has a lower discrimination power than the M-CARS, its accuracy, discrimination, and calibration abilities have acceptable results. Moreover, our study shows that acute stroke (hemorrhagic and nonhemorrhagic) in the first 24 h of CICU admission can impact mortality. Further research can be considered regarding adding acute stroke variables for enhancing the predictive ability of the SCIENCE score. This study demonstrated that the SCIENCE score is a potentially more practical tool in resource-limited settings. However, research on the validity of this score is required in various hospitals, pending broader validation.

### Limitations

First, over 50% of the initial patient cohort was excluded owing to missing AG data, because ABG analysis was not routinely performed in all of our CICU patients. This introduces potential selection bias, as the analyzed population may represent a more critically ill subset, limiting the generalizability of the findings. Second, this study was conducted at a single institution where the SCIENCE score was originally developed, and external validation in other centers or populations has not yet been published. Therefore, to confirm the robustness and applicability of the SCIENCE score, further prospective validation in diverse clinical settings is warranted.

### Conclusion

The M-CARS and SCIENCE score demonstrate acceptable predictive performance for patients in the CICU. However, the SCIENCE score may serve as an alternative for predicting CICU and in-hospital mortality owing to the limited applicability of the M-CARS in certain contexts (particularly in resource-limited hospitals or settings where complete laboratory data, including AG, are not routinely obtained). Moreover, the parameters used in the SCIENCE score are simpler to apply, making it a practical choice for clinicians.

## Supplementary Material

Suppl 1 The M-CARS scoring.

Suppl 2 The SCIENCE scoring.

Suppl 3 Multivariate analysis test of CICU and in-hospital mortality outcomes.

## Data Availability

The data supporting the findings of this study are available from the corresponding author upon reasonable request.

## Abbreviations

ACS: acute coronary syndrome
AG: anion gap
AUC: area under the curve
CI: confidence interval
CICU: cardiovascular intensive care unit
M-CARS: Mayo Cardiac Admission Risk Score
NPV: negative predictive value
PPV: positive predictive value
ROC: receiver operating characteristic
SCIENCE score: Sardjito Cardiovascular Intensive Care score
