# Supplementary material for: Sardjito Cardiovascular Intensive Care Score as an Alternative to Mayo Cardiac Admission Risk Score for Predicting Mortality in Cardiovascular Intensive Care Patients
Source: Cardiol Res. 2026 Jun 5;17(3):170–80. doi: 10.14740/cr2199 (PMC13278723; doi:10.14740/cr2199)
Supplement: Suppl 1 — The M-CARS scoring. [file cr-17-03-170-s001.docx]

**Suppl 1.** The M-CARS Scoring

| Variable |  | Point |  | | |
| --- | --- | --- | --- | --- | --- |
| Admission value of BUN | >23 mg/dL | 1 | **T**  **O**  **T**  **A**  **L**  **P**  **O**  **I**  **N**  **T** | <4 | Low Risk |
|  | ≤23 mg/dL | 0 |  |  |  |
| Admission value of anion gap | >14 | 1 |  |  |  |
|  | ≤14 | 0 |  |  |  |
| Admission Braden skin score | ≤12 | 2 |  |  |  |
|  | 13-15 | 1 |  |  |  |
|  | >15 | 0 |  |  |  |
| Admission value of RDW | >14,3 | 1 |  |  |  |
|  | ≤14,3 | 0 |  |  |  |
| Admission diagnosis of cardiac arrest | Yes | 2 |  |  |  |
|  | No | 0 |  | ≥4 | High Risk |
| Admission diagnosis of shock | Yes | 2 |  |  |  |
|  | No | 0 |  |  |  |
| Admission diagnosis of respiratory failure | Yes | 1 |  |  |  |
|  | No | 0 |  |  |  |
| BUN: blood urea nitrogen, RDW: red blood cell distribution width. | | | | | |
