# Supplementary material for: Sardjito Cardiovascular Intensive Care Score as an Alternative to Mayo Cardiac Admission Risk Score for Predicting Mortality in Cardiovascular Intensive Care Patients
Source: Cardiol Res. 2026 Jun 5;17(3):170–80. doi: 10.14740/cr2199 (PMC13278723; doi:10.14740/cr2199)
Supplement: Suppl 2 — The SCIENCE scoring. [file cr-17-03-170-s002.docx]

**Suppl 2.** The SCIENCE Scoring

| Variable | Point |  | | |
| --- | --- | --- | --- | --- |
| Gender: Female | 1 | **T**  **O**  **T**  **A**  **L**  **P**  **O**  **I**  **N**  **T** | <3 | Low risk |
| Admission diagnosis of acute heart failure | 1 |  |  |  |
| Admission diagnosis of hemodynamic instability | 2 |  | ≥3 | High risk |
| Admission diagnosis of comorbid pneumonia | 1 |  |  |  |
| Serum Creatinine ≥1.5mg/dL | 1 |  |  |  |
| TAPSE <17 mm | 1 |  |  |  |
| Use of Invasive Mechanical Ventilation | 1 |  |  |  |
| TAPSE: Tricuspid Annular Plane Systolic Excursion. | | | | |
