# Supplementary material for: Sardjito Cardiovascular Intensive Care Score as an Alternative to Mayo Cardiac Admission Risk Score for Predicting Mortality in Cardiovascular Intensive Care Patients
Source: Cardiol Res. 2026 Jun 5;17(3):170–80. doi: 10.14740/cr2199 (PMC13278723; doi:10.14740/cr2199)
Supplement: Suppl 3 — Multivariate analysis test of CICU and in-hospital mortality outcomes. [file cr-17-03-170-s003.docx]

**Suppl 3.** Multivariate analysis test of CICU and in-hospital mortality outcomes

| **Variable** | | **P value** | **OR** | **CI 95%** | |
| --- | --- | --- | --- | --- | --- |
|  |  |  |  | **Lower** | **Upper** |
|  | Multivariate analysis test of CICU mortality outcomes | | | | |
|  | M-CARS score | **<0.001*** | 4.772 | 3.536 | 6.441 |
|  | SCIENCE score | **<0.001*** | 2.338 | 1.691 | 3.232 |
|  | Diabetes mellitus | 0.059 | 0.771 | 0.588 | 1.010 |
|  | Sepsis | 0.823 | 1.072 | 0.584 | 1.969 |
|  | Acute stroke | **0.006*** | 1.991 | 1.213 | 3.268 |
|  | LVEF (≤40%) | **0.005*** | 1.492 | 1.126 | 1.978 |
|  | LVEF (41-49%) | 0.123 | 1.340 | 0.924 | 1.944 |
|  | Renal replacement therapy | 0.095 | 1.550 | 0.926 | 2.596 |
|  | Multivariate analysis test of in-hospital mortality outcomes | | | | |
|  | M-CARS score | **<0.001*** | 4.577 | 3.459 | 6.056 |
|  | SCIENCE score | **<0.001*** | 2.270 | 1.682 | 3.062 |
|  | Age (≥60 years) | 0.101 | 1.228 | 0.961 | 1.569 |
|  | Acute coronary syndrome | 0.137 | .819 | 0.630 | 1.066 |
|  | Sepsis | 0.751 | 1.110 | 0.582 | 2.117 |
|  | Stroke | **<0.001*** | 2.502 | 1.514 | 4.134 |
|  | LVEF (≤40%) | 0.177 | 1.208 | 0.918 | 1.588 |
|  | LVEF (41-49%) | 0.761 | 1.058 | 0.735 | 1.522 |
